# Characteristics, likelihood and challenges of road traffic injuries in China before COVID-19 and in the postpandemic era

**DOI:** 10.1057/s41599-022-01482-0

**Published:** 2023-01-03

**Authors:** Ping Yuan, Guojia Qi, Xiuli Hu, Miao Qi, Yanna Zhou, Xiuquan Shi

**Affiliations:** 1 Department of Epidemiology and Health Statistics, School of Public Health, Zunyi Medical University, 563006 Zunyi, Guizhou China; 2 Center for Injury Research and Policy & Center for Pediatric Trauma Research, The Research Institute at Nationwide Children’s Hospital, The Ohio State University College of Medicine, Columbus, OH USA

**Keywords:** Health humanities, Education

## Abstract

Through a review of previous studies, this paper analysed the epidemiological characteristics and attempts to determine the various trends of road traffic injuries (RTIs) in China before and after the coronavirus disease 2019 (COVID-19). This paper proposed effective measures and suggestions for responding to RTIs in China. Moreover, this paper aimed to provide some references for studies on RTIs in the future. According to a reference review, 50 articles related to RTIs were published and viewed in the China National Knowledge Infrastructure (CNKI), Wanfang database, Weipu (VIP) database and PubMed/MEDLINE database. Articles were selected according to the exclusion and inclusion criteria and then classified and summarized. Regarding cases, RTIs in China were highest in summer, autumn, and in rural areas and lowest in February. Men, elderly individuals and people living in rural areas were more susceptible to RTIs. In addition, thanks to effective and proactive policies and measures, the number of RTIs and casualties in China has substantially decreased, while there has been a growing number of traffic accidents along with the increase in nonmotor vehicles. However, it is worth noting that the number of RTIs obviously fell during the COVID-19 pandemic due to traffic lockdown orders and home quarantine policies. Nevertheless, accidents related to electric bicycles increased unsteadily because of the reduction in public transportation use at the same time. The factors that cause RTIs in China can be divided into four aspects: human behaviours, road conditions, vehicles and the environment. As a result, measures responding to RTIs should be accordingly proposed. Moreover, the road traffic safety situation in developing countries was more severe than that in developed countries. RTIs in China showed a downward trend attributed to road safety laws and various policies, and the downward trend was more significant during the COVID-19 pandemic owing to traffic lockdowns and home quarantine measures. It is urgent and necessary to promote road traffic safety, reduce injuries, and minimize the burden of injuries in developing countries.

## Introduction

**Fig. 1:**
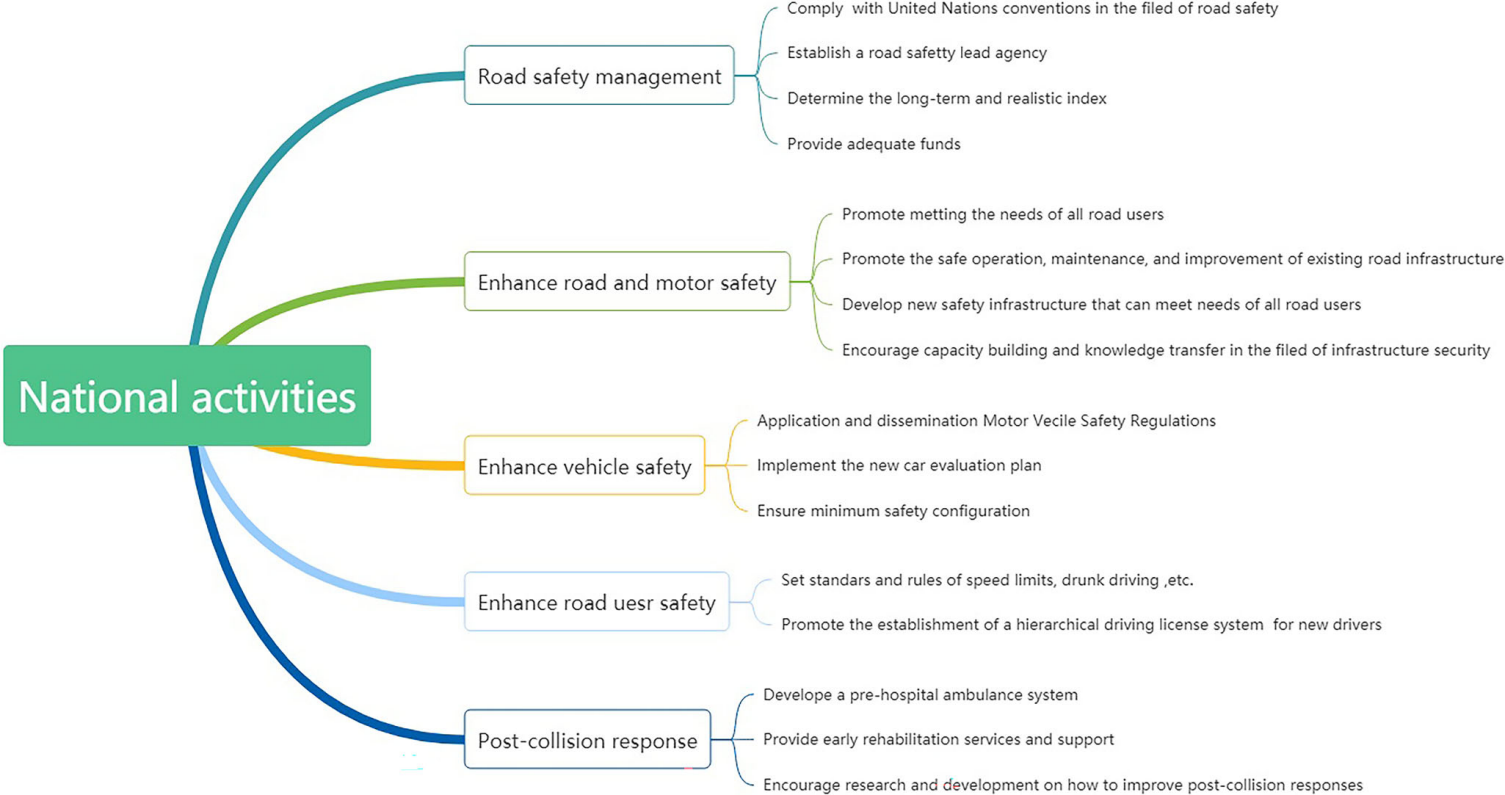
Five pillars of national activites encouraged in the Decade of Road Safety.

Road traffic injuries (RTIs) refer to fatal or nonfatal injuries caused by the collision of at least one moving vehicle on a public road. At present, RTIs are the eighth leading cause of death in the world and the fifth leading cause of reduced life expectancy and have been a persistent problem that the Chinese government is committed to dealing with. As the World Health Organization’s (WHO) Global status report on road safety noted, the number of RTIs worldwide continues to rise, with an increase of ~100,000 in just three years. In 2018, a study indicated that there were ~1.35 million deaths attributed to RTIs every year that seriously threaten the safety of human life and property, and almost 90% of the deaths occurred in low- and middle-income countries (WHO, 2018). The WHO predicted that RTIs may become the fifth leading cause of death worldwide in 2030. Therefore, the United Nations (UN) proposed a Decade of Action for Road Safety, which aimed to halve the number of casualties caused by RTIs by 2020 (Pérez-Núñez et al., 2021). Over the past decade, members of the UN have pledged to undertake actions to promote road safety and enhance road safety management by such actions as developing and enforcing legislation regarding key RTI risk factors (such as limiting speed, reducing drunk driving, and increasing the use of seat belts, child restraints and motorcycle helmets); improving road and vehicle safety standards; and promoting road safety and enhancing road safety management (Peden, 2010). The actions in the theoretical framework are shown in Fig. 1. Considering the seriousness of RTIs worldwide, the WHO and the UN have called on all countries to address road traffic safety and take effective measures to prevent RTIs. China is the largest developing country in the world that has the longest high-speed roads. In China, RTIs have become the primary cause of injury-related casualties (Zhang et al., 2011). According to Global Burden of Disease (GBD) study data, China’s road traffic deaths accounted for 21% of global road traffic deaths in 2017, and the number was ~262,000 (Wang et al., 2019). The main cause of RTIs is a large number of motor vehicles. RTIs can also give rise to serious economic losses, and the total economic cost of RTIs in China was calculated as 490.1 billion RMB in 2017, which was equivalent to 0.60% of the GDP (gross domestic product, GDP) (Tan et al., 2020).

Although some studies have reported that RTIs in China have shown a downward trend, the mortality of road traffic accidents is still much higher than that in developed countries. Additionally, traffic control during the COVID-19 epidemic period substantially improved China’s road traffic safety, but we cannot reduce RTIs by restricting road traffic. In the post-COVID-19 pandemic era, the implementation of road traffic safety is also a major challenge that China needs to tackle as a result of the rapid development of China’s economy and society.

The main purpose of this review was to clarify the trend of RTIs before and after the COVID-19 pandemic and the main reasons for the change in RTIs in China, particularly exploring the impact of China’s road traffic safety law, traffic lockdowns during the COVID-19 and home quarantine policies on RTIs. Moreover, it is essential to summarize the primary factors of RTIs in China and develop suggestions to formulate preventive measures to reduce the burden of RTIs in China and other similar countries.

## Methods

### Search strategy

In November 2021, the literature was searched in the following electronic databases: China National Knowledge Infrastructure (CNKI), Wanfang database, Weipu (VIP) database and PubMed/MEDLINE. Then, Chinese and English language documents published in the research field of RTIs in China were collected. First, the keywords in Chinese ((“road traffic injury” OR “traffic accidents”) AND (“epidemiology” OR “characteristics”)) or ((“traffic injury OR traffic safety”) AND “COVID-19”) were used to search the CNKI, which yielded 87 documents and 2 documents in Chinese and English, respectively. When using the same search keywords as those used in the CNKI, 147 articles and 281 articles were obtained from the Wanfang database and VIP database, respectively. In the MEDLINE database, 414 articles were obtained by using the keywords ((“road traffic injury” OR “road traffic accident” OR “car accident”) AND (“epidemiological characteristics” OR “risk factors” OR “variation tendency” OR “COVID-19”)”. A total of 931 articles were collected from these databases.

### Inclusion and exclusion criteria

Whether a paper was eligible for inclusion was determined by reading the abstract. The study inclusion and exclusion criteria were as follows (Chen et al., 2014): (1) Inclusion criteria: articles about RTIs published up to December 31, 2021, that described the trend or characteristics of RTIs in China; (2) Exclusion criteria: articles that discussed other diseases caused by RTIs; duplicate articles and articles without full text. Finally, a total of 50 eligible articles about RTIs were included after reviewing the literature.

## Results

### RTIs in China

China is the most populous developing country in the world. Motorization has become an irreversible trend in China, resulting in increasingly severe traffic accidents. There were ~384 million motor vehicles in China in 2019, of which 260 million were cars (Ma et al., 2020). At the end of 2016, China’s investment in transportation exceeded 5 trillion RMB, and the total road mileage increased from 1.87 million kilometers in 2004 to 4.7 million kilometers in 2016, with an increase of 151.05% in these years (Sun et al., 2019). The “Outline of the Healthy China 2030 Plan” was proposed by the Chinese government and included a 30% reduction in deaths caused by traffic accidents in China by 2030. Researchers also found that China’s RTIs mainly occurred in developed provinces such as Zhejiang, Jiangsu, and Anhui (Wang et al., 2018), which may be related to the level of economic development. Although a number of studies have shown that RTIs in China have generally presented a downward trend in general, the burden of disease caused by RTIs is still a serious problem. Moreover, the safety of nonmotor vehicles should also be strengthened as a result of the popularity of bicycle sharing (Ye et al., 2019).

### Epidemiological characteristics of RTIs

Generally, RTIs in China mostly occur in certain seasons and periods. A project in Beijing found that accidents mostly occurred in months with higher temperatures, such as in summer and autumn, and traffic injuries accounted for the largest proportion of all injuries (Zhu et al., 2020). In addition to Beijing, other regions also had similar time distribution characteristics, and almost every study showed that the fewest traffic accidents occurred in February (Zhu et al., 2019; Pan et al., 2016). In addition, most traffic accidents occurred between 8:00–9:00 and 16:00–17:00. However, the highest number of fatalities occurred in the early morning between 02:00 and 05:59 (Wang et al., 2019).

From 1990 to 2013, the total death rate of RTIs in China increased by 0.54 per 100,000 people, and the rate among males rose by 2.34 per 100,000 people. Additionally, the mortality rate, YLLs (years of life lost, YLLs) and YLDs (years lived with disability, YLDs) of males were higher than those of females (Wang et al., 2017). According to a study in Guangdong Province, China, people who are 20–59 years old have the greatest risk of death from RTIs, and the mortality rate of men is generally higher than that of women (Zhang et al., 2020). In addition, pedestrians account for 50% of the death toll in RTIs. This survey mainly referred to men, and elderly individuals in Jiangsu Province were more likely to be involved in RTIs (Ding et al., 2017). At the same time, RTIs and drowning were the two major causes of death among teenagers in China (Yin et al., 2021).

RTIs mainly occur on urban general roads and secondary roads, and the injury rate of RTIs in rural areas in China is higher than that in urban areas and in mountainous areas compared with plains areas; most traffic accident casualties in China occur in mountainous areas. When analysing traffic accidents by subregion, the highest RTI rate was observed in the most economically developed eastern region and the lowest was observed in the western region (Wang et al., 2019).

### The main influencing factors of RTIs

#### Human factors

According to previous research data, most RTIs are caused by the driver’s behaviours (speeding, drunk driving and not wearing a seat belt). Private vehicles sometimes disregard traffic signs and traffic lights in daily life (Ambo et al., 2021). In addition, the driver’s personality and psychological characteristics are also related to the occurrence of RTIs. For instance, an outgoing personality, hostility and fear are the main risk factors for drivers involved in traffic accidents (Su et al., 2014). Pedestrians are the most common victims of RTIs. In China, due to violations of traffic rules, there are 600–800 accidents caused by pedestrians violating traffic signals each year, and the number of deaths is ~200–300 people (Hua et al., 2019); in addition, such behaviours as children suddenly running onto the road, young people crossing the road, and elderly individuals suddenly turning back in the middle of the road are other reasons for RTIs.

#### Vehicle factors

At present, China’s car sales are the highest worldwide. Although vehicle safety performance and in-vehicle safety protection measures are critically evaluated, safety incidents are often caused by automobile breakdowns; for example, people’s attention to vehicle safety has been improved by brake failure accidents of some vehicles. Until 2016, there were 250 million registered electric vehicles in China according to police statistics, and the number of deaths resulting from electric vehicle accidents increased 11-fold from 2004 to 2015 (Zhang et al., 2018).

#### Road factors

China’s investment in transportation is increasing yearly. Economic development promotes road construction; as a result, the number of casualties from RTIs increases by 284.04 for every additional 10,000 kilometres of road mileage (Sun et al., 2019). Rural areas lack adequate funding for road construction investment and have poor-quality road infrastructure and insufficient traffic signal lights and road markings, which frequently cause traffic accidents in countryside areas. Currently, mixed traffic is the main feature of urban areas in China (Zhao et al., 2018). Unreasonable urban road design and other factors cause traffic accidents, such as insufficient road capacity and road branches to divert traffic.

#### Environmental factors

Severe weather, rain, snow and fog often lead to serious traffic accidents. Factors such as sunshine duration, temperature and wind speed were all significantly correlated with RTIs according to research, and wind speed was negatively correlated in Shantou city, Guangdong Province (Gao et al., 2016). Another study demonstrated that the relative risk of accidents increased under severe weather conditions, and the risk of traffic accidents on expressways was substantially higher than that on two-lane and multilane roads when the weather was poor (Malin et al., 2019). Studies have also shown that both high and low temperatures can substantially increase the number of traffic accidents (Zhan et al., 2020; Liang et al., 2021).

### RTIs in China before and after the COVID-19 pandemic

**Fig. 2:**
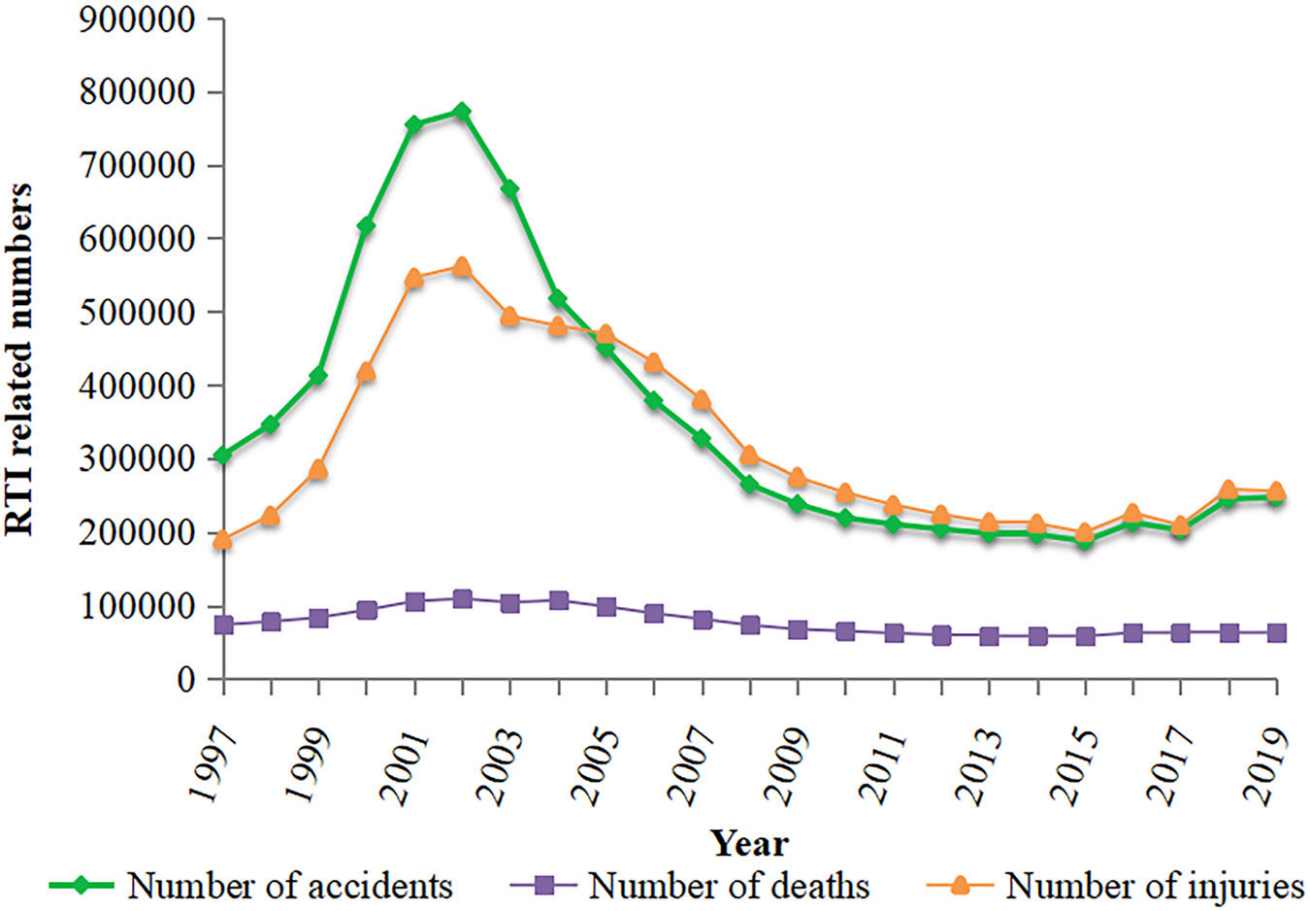
Trends in the number of road traffic injures in China from 1997-2019.

According to research data on RTIs from 1997 to 2016 (Ye et al., 2019) and data on RTIs on the official website of the Ministry of Transport of China from 2017 to 2019, a line chart was drawn, as shown in Fig. 2 (its data source can be found in Table 1), which clearly shows the downwards trend of RTIs in China. Both the number of accidents and casualties slowly decreased, with a peak in 2002, and then declined in ~2010; however, traffic accidents increased considerably in 2016. The reason might be attributed to the prevalence of shared bicycles, which increased the probability of collision with motor vehicles. Although bicycle sharing is greatly convenient, traffic accidents have increased more rapidly due to passengers on shared bicycles than lone riders on bicycles.

**Table 1:** Data source.

Time (year)	Reference or data source	Information authority level
Figure 2		
1997–2016	China National Knowledge Infrastructure (CNKI). http://162.14.77.248/kns/brief/Default_Result.aspx?code=SCDB	Official
2017–2019	Nation Bureau of Statistics. http://www.stats.gov.cn/tjsj/ndsj/	Official
Figure 3		
2010, 2013	PubMed. https://www.ncbi.nlm.nih.gov/books/NBK361926/	Official

Although China’s traffic system has developed rapidly in a short period of time and motorization has caused many casualties, the downward trend in RTIs in China in recent years looks optimistic. Traffic laws and regulations are important to limit road users, and China issued the China Road Traffic Safety Law in 2004 and revised it in 2011 (Fayard, 2019). One previous study confirmed that China’s road traffic safety has improved as a result of the more stringent drinking and driving law revised in 2011 (Fei et al., 2020). The road traffic safety law issued by the government stipulates that drivers and passengers should use seat belts, not drive after drinking, drive at the speed limit, wear safety helmets during riding and adopt other protective measures; in addition, the corresponding departments should punish illegal behaviours (Fayard, 2019). In addition to policy and legal constraints, the external environment is conducive to reducing RTIs. In China, surveillance cameras promoting the enforcement of traffic laws are installed in every city and are mainly used to detect illegal behaviours during driving to reduce the risk of collisions, such as speeding and running red lights (Wang et al., 2020). In summary, stricter enforcement of laws and various preventive measures are the main reasons for the reduction in RTIs in China.

During the COVID-19 pandemic, the incidence of traffic accidents in China dropped considerably due to traffic lockdowns and home quarantine. A survey showed that during the period of traffic lockdowns caused by COVID-19, the number of traffic accidents per week was at its lowest point during the implementation of traffic control in Ningbo city. RTIs gradually increased and stabilized to 30–40% compared with the same period in 2019 after the resumption of work and production (Zhao et al., 2020). Moreover, the results of the analysis of casualties during the period of COVID-19 in Suzhou suggested that the total death rate caused by injuries and the number of RTIs were significantly lower than those in previous years, while the rates of drowning and poisoning exceeded the normal fluctuation range (Wei et al., 2021), which was consistent with a previous study conducted in Suzhou city. The results showed that the number of RTIs during the period of the COVID-19 pandemic decreased (Huang et al., 2021).

In addition, a recent study suggested that the death rate caused by traffic injuries was lower than that of suicide and falls during the pandemic in Wuhan city (Liu et al., 2021). Researchers reported that the number of RTIs dropped sharply with the implementation of the community quarantine policy in the early stage of the outbreak of COVID-19 and increased gradually due to economic recovery in the later period in Anhui Province (Zhu et al., 2020). Meanwhile, the decline in the incidence of fractures among Chinese children during the COVID-19 pandemic was also related to traffic restrictions and reductions in RTIs (Li et al., 2021). However, the probability of accidents on private transportation vehicles was still high due to the increasing number of vehicles during the COVID-19 pandemic in Taiwan, China; as a result, the death toll increased slightly, which was significantly different from mainland China (Gao et al., 2021).

This phenomenon was also seen in other countries. For example, a previous study demonstrated that the implementation of traffic blockades, telecommuting and online teaching measures during the COVID-19 period significantly reduced the casualties caused by RTIs in India (Jain et al., 2020); moreover, studies confirmed that although minor or nonfatal accidents were reduced by traffic blockades during the COVID-19 pandemic, serious injuries and car accidents did not decrease substantially in the United States (Qureshi et al., 2020), even though the number of fatal accidents increased during the lockdown (Adanu et al., 2021).

Strictly speaking, the number of RTIs that occurred in China during the COVID-19 pandemic fell, but not directly due to COVID-19. After the outbreak of COVID-19, a correct epidemic prevention policy was formulated quickly by the government, and home quarantine and traffic lockdowns were carried out nationwide; as a result, the traffic flow in cities and streets decreased rapidly. For example, the traffic flow in Beijing dropped by 46.9% during the traffic blockade, and as a result, vehicle collisions were greatly reduced (Xin et al., 2021). In summary, traffic blockades during the COVID-19 epidemic were conducive to reducing the incidence of RTIs and property losses caused by traffic accidents.

### Challenges of RTIs in China

Reducing RTIs in China, the largest developing country in the world is a tremendous challenge. On the one hand, there was a downward trend in RTIs during the COVID-19 period, which cannot be fundamentally explained by some of the measures that were implemented in response to COVID-19. On the other hand, it is key that the government reduce the loss of life and property caused by RTIs.

The effectiveness of RTI prevention and control efforts is different between developed and developing countries. Because more attention is given to illegal activities, such as not wearing seat belts and speeding, the number of deaths caused by RTIs in the United States and other developed countries is slowly falling. The number of deaths from RTIs was reported to be 5% less each year, and the number of serious injuries was more than 50% less in India (Jagnoor et al., 2015). Moreover, the mortality rate of traffic accidents was 26.6 per 100,000 people in Africa (Adeloye et al., 2016), and the countries that implemented RTI interventions were mainly located in East Africa and Southern Africa. The reason might be that there were no systematic intervention measures on road traffic in other regions (Bonnet et al., 2018).

**Fig. 3:**
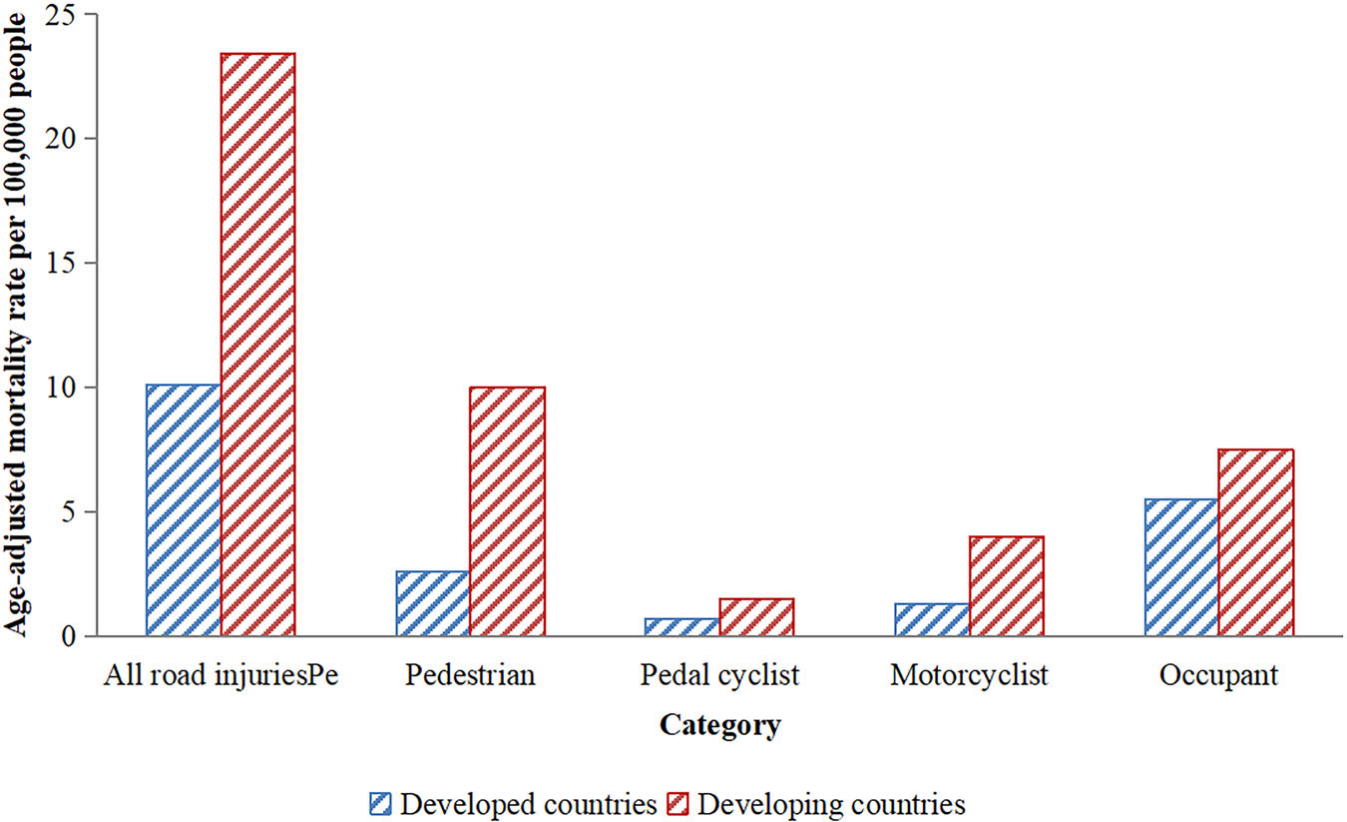
Age-adjusted road injury death rate per 10,000 population by development statues and type of road users inthe Decade of Action for Road Safety.

The WHO reported that approximately 1.35 million people die from RTIs every year, 90% of which occur in developing countries (WHO, 2018), and another study showed that the economic losses caused by RTIs in low- and middle-income countries account for more than 5% of GDP (Dhibi, 2019). According to the results of a previous study, the age-adjusted mortality rate among different types of road users in developed and developing countries was compared during the Decade of Action for Road Safety project (Ning et al., 2016). The results suggested that the mortality rate in developing countries was higher than that in developed countries regardless of the type of road users, and pedestrians were the most vulnerable people (see Fig. 3 (its data source can be found in Table 1)). It is urgent for developing countries to strengthen road traffic safety to reduce the disease burden of RTIs.

## Discussion

Although studies (Ye et al., 2019; Wang et al., 2019) have reported that China’s RTIs are showing a downward trend, the burden of RTIs in China is still a serious problem. Additionally, the results of the trend map of China’s RTIs indicated that there was a downward trend for the first time in 2003 after a peak in 2002, which very likely coincided with the SARS epidemic, without any further evidence to prove its universal importance. To some extent, the main reason for the decline in RTIs in China could be due to the enforcement of the Road Traffic Safety Law and the strict implementation of various law enforcement actions. However, the increase in traffic accidents in 2016 might be due to the sudden increase in shared bicycles, which could have increased the probability of collisions with motor vehicles. In general, more attention should be given to accidents caused by nonmotor vehicles, which have gradually become a serious public health problem.

In addition, males in China are more likely to suffer RTIs, which might be the result of the larger number of male drivers. Personality characteristics and driving behaviours among men are distinct from those among women, which may be related to more RTIs among males (Wang et al., 2017; Zhang et al., 2020). In addition, illegal driving behaviours such as drunk driving and speeding are more likely to occur among men; regarding the higher number of cases in summer and autumn, a series of research results (Zhu et al., 2020, 2019; Pan et al., 2016) indicated that a great number of people travel during these seasons with a sharp increase in traffic volume and RTIs. More people staying at home during the Spring Festival (Chinese New Year), usually in February, resulted in the lowest number of RTIs in China. Moreover, the probability of serious traffic accidents might increase due to rainy and snowy weather. Traffic lockdowns during the COVID-19 pandemic in February greatly decreased the number of cases in China to some extent. Although the United States implemented a traffic blockade, speeding was more severe, and the probability of serious RTIs was much greater due to the lack of traffic control. Additionally, crashes and collisions occurred more frequently during peak hours, especially morning and evening; before sunset, when there are fewer vehicles on the road, speeding may lead to more serious RTIs. Moreover, the occurrence of accidents in the western region was more frequent than that in any other region due to its mountainous terrain, which is complex and difficult to navigate. In addition, RTIs in rural areas occurred more frequently than in urban areas because of poor road construction and less safety awareness among people. As a result, it is imperative for authorities to improve road conditions in rural areas.

Compared with developed countries, developing countries are in a stage of economic upswing, accompanied by an increased number of motor vehicles, but in fact, there is no relatively complete transportation system that leads to frequent RTIs. There are sufficient funds for road safety in developed countries, and these countries attach great importance to road safety, which is conducive to the reduction of RTIs in some countries; therefore, there is a large gap in RTIs between developed and developing countries. Only when traffic safety in developing countries is improved can the burden of disease and property losses caused by global traffic injuries be relieved.

There is no relatively complete traffic management system in China. Moreover, penalties for traffic violations in China are still lacking compared to those in developed countries, especially for problems such as drivers and pedestrians that disregard traffic rules and their poor walking and driving behaviours. As a result, traffic injuries caused by human factors can be greatly alleviated by implementing appropriate measures.

Currently, we can take many measures to fight against RTIs. First, laws need to be further enforced. Most RTIs are caused by the direct behaviours of drivers due to their poor safety awareness and illegal actions. The evaluation and supervision of drivers, especially motorcycle drivers, must be strengthened to improve the awareness of road traffic safety (Wang et al., 2015). At the same time, law enforcement is the most effective strategy for driving behaviour, especially regarding speed limits and drunk driving (Aguilera et al., 2014). In addition, it is essential to use seat belts and wear a safety helmet.

Second, it is necessary to improve vehicle safety performance and examine vehicle safety frequently. In addition, regular road, traffic signal and road safety sign inspections should be conducted. Finally, satellite navigation can play an important role in reducing traffic injuries to some extent. China’s Beidou Navigation Satellite System (BDS), which has good performance and accurate positioning, is one of the four major satellite navigation systems in the world and can detect bad driving behaviours, such as speeding and fatigued driving in time and can even issue alarm signals to remind the driver to adhere to safe driving behaviours.

Reducing the burden of RTIs in China will be long and difficult. Given that, the following are suggested. First, it is of great importance to improve comprehensive road weather monitoring to predict road weather, especially on expressways. Additionally, with the development of driverless technology, there is still a lack of research on the road traffic system required by unmanned technology.

In addition, RTIs caused by patients with mental disorders and users of psychotropic substances have gradually increased, so it is essential to strengthening the management and control of patients with mental disorders. However, people with mental disorders should not be allowed to apply for a motor vehicle driving licence in China; as a result, it is necessary to establish a linked working mechanism between the medical system and the traffic control department. Last, data management should be further improved to not only show trends in RTIs but also play a pivotal role in preventing and reducing traffic accidents.

## Conclusion

In summary, both the number and rate of RTIs in China significantly declined during the COVID-19 pandemic, which was closely related to traffic blockades during the pandemic. Preventive and protective measures for traffic safety have been put forwards to promote road traffic safety based on human factors, road factors, vehicle factors and environmental factors. In addition, further enforcement of laws and regulations on nonmotor vehicle driving should also be improved in China. However, collaboration among countries, especially developing countries, to reduce the global burden of RTIs remains a challenge.

## Data Availability

This article does not analyse or generate any datasets.
